# Human NK Cell Subsets Redistribution in Pathological Conditions: A Role for CCR7 Receptor

**DOI:** 10.3389/fimmu.2016.00414

**Published:** 2016-10-07

**Authors:** Silvia Pesce, Lorenzo Moretta, Alessandro Moretta, Emanuela Marcenaro

**Affiliations:** ^1^Dipartimento di Medicina Sperimentale, Università degli Studi di Genova, Genova, Italy; ^2^Dipartimento di Immunologia, IRCCS Bambino Gesù Ospedale Pediatrico, Rome, Italy; ^3^CEBR, Università degli Studi di Genova, Genova, Italy

**Keywords:** human NK cells, CCR7, transplantation, alloreactivity, NK cell subsets, immune checkpoint

## Abstract

Innate and adaptive immunity has evolved complex molecular mechanisms regulating immune cell migration to facilitate the dynamic cellular interactions required for its function involving the chemokines and their receptors. One important chemokine receptor in the immune system is represented by CCR7. Together with its ligands CCL19 and CCL21, this chemokine receptor controls different arrays of migratory events, both in innate and adaptive immunity, including homing of CD56^bright^ NK cells, T cells, and DCs to lymphoid compartments, where T cell priming occurs. Only recently, a key role for CCR7 in promoting CD56^dim^ NK cell migration toward lymphoid tissues has been described. Remarkably, this event can influence the shaping and polarization of adaptive T cell responses. In this review, we describe recent progress in understanding the mechanisms and the site where CD56^dim^ KIR^+^ NK cells can acquire the capability to migrate toward lymph nodes. The emerging significance of this event in clinical transplantation is also discussed.

## Introduction

NK cells are effector cells of the innate immune system able to recognize and kill stressed, transformed, or virus-infected cells *via* a delicate balance of signals transmitted by activating and inhibitory receptors, and to secrete various effector molecules (1–3).

Two main subsets of human NK cells have been identified, according to the cell surface density of CD56 and expression of CD16 (FcγRIIIa). The CD56^dim^ CD16^bright^ NK cell subset expresses KIR and/or CD94/NKG2A molecules and predominates in peripheral blood (~90% of circulating NK cells), while the CD56^bright^ CD16^neg/dim^ NK cells express CD94/NKG2A (but are KIR negative) and represent only ~10% of circulating NK cells. CD56^dim^ CD16^bright^ NK cells display potent cytolytic activity and produce cytokines following receptor-mediated stimulation (e.g., engagement of activating surface receptors during target cell recognition) (4–6). On the other hand, CD56^bright^ CD16^neg/dim^ NK cells produce cytokines including interferon-γ (IFNγ), tumor necrosis factor-α (TNFα), and granulocyte–macrophage colony-stimulating factor (GM-CSF) and undergo proliferation following stimulation with pro-inflammatory cytokines. Cytolytic activity is acquired only after prolonged cell stimulation (4–6). Notably the CD56^bright^ CD16^neg/dim^ NK cells can undergo differentiation into CD56^dim^ CD16^bright^ NK cells. Moreover this subset can undergo further phenotypic and functional maturation toward terminally differentiated NK cells (7–10).

## What Determines NK Cell Subset Recruitment to Different Organs During Physiological and Pathological Conditions?

In bone marrow, NK cell precursors undergo a maturation process that includes the acquisition of effector functions and the expression of chemotactic receptors that will drive their migration from the bone marrow to different organs through the blood stream (11, 12).

The recirculation and the distribution of cells of the immune system to the various organs depend primarily on the release of particular chemokines by organ-specific cell types (13, 14).

NK cells can respond to a large array of chemokines (13), and can be recruited to different district of the body and to sites of inflammation (15, 16). The distribution of NK cells is subset specific. Indeed, the two main NK cell subsets display major functional differences not only for their cytolytic activity and modality of cytokine production but also in their homing capabilities, as shown by their organ-specific localization (16). In particular, the cytolytic CD56^dim^ CD16^bright^ NK cell subset expresses CXCR1, CX3CR1, and ChemR23 chemokine receptors; therefore, it is mainly recruited to inflamed peripheral tissues. In contrast, CD56^bright^ CD16^neg/dim^ NK cells preferentially express CCR7 and are primarily attracted by secondary lymphoid organs (lymph nodes, tonsils, and spleen) (17–20). These cells also express CD62L (L-selectin), which provides important adhesion to endothelial surfaces, required for extravasation of CD56^bright^ NK cells (21). Accordingly, CD56^bright^ NK cells are 10 times more frequent than CD56^dim^ in parafollicular (T-cell) regions of healthy (non-inflamed) lymph nodes, where they can be activated by T-cell-derived IL-2 (19, 22). Therefore, it is likely that the expression of the high-affinity IL-2 receptors on CD56^bright^ NK cells may promote a cross talk between NK and T cells in these lymphoid compartments (19).

It has recently been shown that, in addition to secondary lymphoid compartments (SLCs), CD56^bright^ CD16^neg/dim^ NK cells populate other normal human tissues. These include uterine mucosa, liver, skin, adrenal gland, colorectal, liver, and visceral adipose tissues. On the other hand, tissues such as lung, breast, and sottocutaneous adipose tissue contain preferentially CD56^dim^ CD16^bright^ cells (14, 16, 23).

The specific distribution of the two subsets is mainly reflecting differences in their chemokine receptor repertoires and, as a consequence, in their ability to respond to different chemotactic factors (14, 16, 23).

Remarkably the localization of the two NK cell subsets can change in pathological conditions, e.g., in the presence of tumors (16). Thus, in different tumor types, both migration and homing of NK cells may be altered and even reversed. For example, NK cells present in tumor-infiltrated peripheral tissues are often enriched in CD56^bright^ CD16^neg/dim^ NK cells (24–26); in contrast, an expansion of an unusual subset characterized by a CD56^dim^ CD69^+^ CCR7^+^ KIR^+^ phenotype has been detected in tumor-infiltrated lymph nodes (27). A possible explanation of these findings is that, a different pattern of chemokines, released by cells of the tumor microenvironment, or the acquisition of different/new chemokine receptors by NK cells, may operate an altered recruitment of the two NK cell subsets.

Thus, chemokines/chemokine receptors play a critical role in the regulation of the distribution of NK cell subpopulations in the various tissues, both in normal and the pathological conditions, such as inflammatory processes or tumors.

Notably, also organ-specific features, such as the anatomical structure, the type of cells present in the tissue, the soluble factors released, and the different interactions that NK cells can establish with different cell types, may considerably influence their homing capability.

## What Conditions Determine NK Cell Activation in Inflamed Tissues?

As illustrated above, in the course of pathological conditions, such as inflammation, viral infection, and tumor growth, NK cells are rapidly recruited from peripheral blood into injured tissues, thanks to the interplay between chemokines and their receptors (20, 28–31).

NK cells, once they reach inflammatory sites, need to be activated in order to perform their functional activities. NK cell activation can occur through different types of signals. These include signals delivered by several activating NK receptors (32), upon recognition of specific ligands expressed on tumor cells, or signals generated in response to stimulation *via* toll-like receptors (TLR) that are constitutively expressed by NK cells and allow their responses to products of viral or bacterial origin (3, 33, 34). In addition, the activation of NK cells can occur through various soluble signals, including different cytokines that are provided by other cell types. Indeed, although NK cells can directly recognize cells infected by viruses and/or transformed cells, many recent studies revealed that the microenvironment and the interaction with other cells of the immune system, in particular with DCs, may considerably contribute to ensure an optimal priming of NK cells. For example, the production of cytokines (such as IL-12) by activated DCs improves NK cell proliferation, production of IFNγ, and antitumor cytotoxic activity (35).

In addition, a full NK cell activation allows the establishment of important processes, such as the mechanism of NK-mediated “editing” of DCs (28), which would play a crucial role in controlling the induction of appropriate antigen-specific adaptive immune responses (36, 37). In particular, activated NK cells appear to contribute to the quality control of immature DCs (iDCs) undergoing maturation by selecting DCs most fitting to optimal antigen presentation (36, 38). This process is thought to be crucial for the subsequent DC migration to SLCs and priming of naïve T lymphocytes toward Type 1 (Th1) immune responses.

In this context, it has to be considered that the exposure of NK cells to type II cytokines, such as IL-4 (35), released by other cells of the innate immune system in certain inflammatory microenvironments may result in low levels of cytotoxicity against tumor cells. Importantly, NK cells would become unable to kill iDCs and to release IFNγ and TNFα. This results in the failure of an efficient “editing” process and in the generation of Th0 cell leading to non polarized T cell responses in lymph nodes that compromise downstream antigen-specific, Th1-mediated immune responses (35). On the other hand, IL-4 by driving a type II immune response may be important for the eradication of particular pathogens. The absence of NK cells driving a Th1 response, in this circumstance, may be optimal for an efficient immune response to that particular pathogen (39).

Other studies suggest that NK cells may play not only a beneficial regulatory role in shaping adaptive immune responses but also an inhibition of the adaptive immune cells (e.g., T and B cells), by killing both antigen-presenting cells (APCs) and antigen-specific T cells and by producing anti-inflammatory cytokines, such as IL-10. These events can shape the overall immune response against certain pathogens, which can have consequences for disease pathogenesis, and infection outcome. Continued evaluation of the specific context-dependent roles of NK cells in human infection will be necessary to guide attempts to modulate NK cells in therapy or prevention of infection (40).

Thus, depending on the nature of the pathogens responsible for the invasion of a given tissue and, as a consequence, on the cytokine microenvironment created during the early stages of an inflammatory response, NK cells may differentially contribute to the quality and magnitude of both innate and adaptive immune responses (1, 35, 41).

## How Do the Phenotypic Properties (And the Effector Functions) of NK Cells Change Following Recruitment to Inflamed Peripheral Tissues?

According to the data reported in the previous paragraphs, there is clear evidence that CD56^dim^ CD16^+^ NK cells, which have been recruited into inflamed tissues, in the presence of pro-inflammatory stimuli favoring their interaction with DCs (28, 31, 42–44) may usually influence naïve T cell priming, for instance by the “DC editing program.” This mechanism may take place in the periphery and, at least in some instances, does not require the recruitment of tissue-activated NK cells into lymph nodes (35, 41, 45).

On the other hand, the inflammatory microenvironment and the interactions established with other cells at the inflammatory sites may also affect both phenotypic and homing properties of NK cells by generating altered NK cell subsets. For example, there is evidence that, during an immune response, the highly cytotoxic CD56^dim^ KIR^+^ (as well as CD56^dim^ KIR^−^ NKG2A^+^) NK cells, that, different from CD56^bright^ NK cells are CCR7 negative, could be directed from the peripheral (non-lymphoid tissues) toward inflamed lymph nodes. These represent a crucial site for NK cell-mediated immunosurveillance against tumor metastases and the control of viral infections and also contribute to priming of adaptive immune responses (27, 41, 46).

In this context, it has been demonstrated that NK cells exposed to a microenvironment rich of IL-18 may *de novo* express CCR7 and, thus, acquire the ability to respond to the lymph node chemokines CCL19 and CCL21 (47).

**Figure 1:**
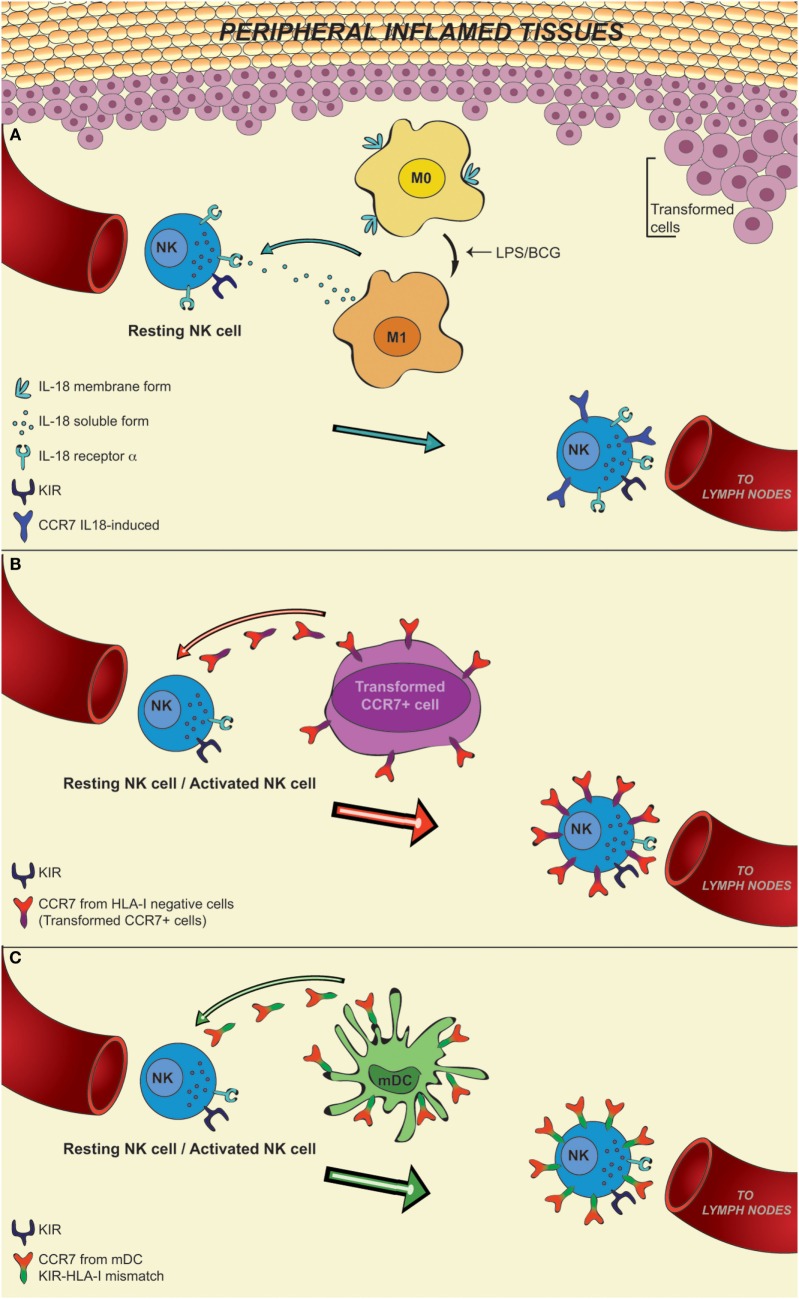
**In this figure, it is shown that CD56^dim^ KIR^+^ NK cells, after recruitment from blood into inflamed peripheral tissues in response to chemokine gradients, may *de novo* express CCR7, a chemokine receptor able to confer immune cells with the ability to migrate to lymph nodes**. This event may occur in a microenvironment rich in IL-18, a pro-inflammatory cytokine released by M0 macrophages during differentiation toward M1 following activation. This *de novo* expression can be induced only on a fraction of NK cells characterized by the expression of the IL-18Rα (resting NK cells), whereas it cannot be induced on activated NK cells that express little IL-18Rα **(A)**. Otherwise, the expression of CCR7 on CD56^dim^ NK cells can be induced by a mechanism of trogocytosis on either resting or activated NK cells, regardless of their level of IL-18Rα expression. This mechanism is finely controlled by the specific interaction between KIRs on NK cells and HLA class I molecules on CCR7^+^ cells. In particular, inhibitory KIRs block this transfer, whereas some activating KIRs are able to strongly promote the CCR7 acquisition by NK cells. This means that in an autologous setting, NK cells may acquire CCR7 only by interacting with HLA-I negative CCR7^+^ cells, such as transformed cells **(B)**. In contrast, in an allogeneic setting, as in haplo-HSCT, characterized by KIR/HLA class I mismatch, KIR^+^ alloreactive NK cells can express CCR7 when they interact with allogeneic CCR7^+^ mDCs **(C)**.

IL-18 may be released by APCs in an inflammatory microenvironment triggered by pathogens. In this regard, it has recently been demonstrated that M0 and M2 macrophages express a membrane form of IL-18 (mIL-18), which is released in the course of polarization to M1, induced by bacterial stimuli (LPS or BCG). IL-18 induces NK cells to release large amounts of IFNγ and plays a pivotal role in the expression on their surface of CCR7 receptor (47, 48) (Figure 1A).

The acquisition of CCR7 provides CD56^dim^ KIR^+^ NK cells with the potential to reach lymph nodes where they can interact with DCs and T cells and regulate T cell responses (41, 47, 49). On the other hand, the expression of CCR7 in response to IL-18 can occur only in resting NK cells, because cytokine-mediated priming of NK cells induces their activation and a marked downregulation of IL-18Rα expression (50, 51). In light of these data, it is conceivable that other mechanisms may contribute to the acquisition of CCR7 by “activated” CD56^dim^ NK cells. Indeed, recent data revealed that an unexpected crosstalk between NK cells and other cells present in the inflamed niche environment (tumor/infected cells or other immune cells, including DCs and T cells) leads to the *de novo* surface expression of CCR7 by CD56^dim^ KIR^+^ NK cells in an IL-18-independent manner (51–54) (Figures 1B,C).

The acquisition of CCR7 by these NK cells requires direct cell-to-cell contact, is detectable within few minutes, and is due to receptor uptake from CCR7^+^ cells. This mechanism is termed “trogocytosis” and is distinct from other mechanisms of intercellular exchanges, such as nanotubes and exosomes (55–58). For example, interaction between NK cells and mature DCs (mDCs) that express surface CCR7 could induce its expression on NK cells (52).

An important aspect is that the acquisition of CCR7 is promoted by non-HLA-specific activating receptors (including NKp46), but, it is negatively regulated by the interaction between inhibitory KIR receptors expressed by NK cells (“acceptor”) and HLA class I molecules expressed by CCR7^+^ cells (“donor”). In particular, in the course of infection in a self-environment, KIR^+^ NK cells can acquire CCR7 only when they interact with HLA class I negative CCR7^+^ cells (e.g., target cells that have undergone tumor transformation or viral infection) (Figure 1B). On the contrary, in an allogeneic setting characterized by KIR/HLA class I mismatch, KIR^+^ NK cells can acquire CCR7 following interaction with any CCR7^+^ cell, including those expressing high levels of HLA class I, such as mDCs (Figure 1C).

This condition is reproduced during KIR-mismatched haploidentical hematopoietic stem cell transplantation (haplo-HSCT) to cure high-risk leukemias (46, 59). Importantly, these events have been recently reproduced and confirmed *in vivo* in a mouse model (53).

Thus, the *de novo* CCR7 expression renders CD56^dim^ KIR^+^ NK cells able to migrate into the SLCs in response to CCL19 and CCL21 chemokines (47, 52, 54). In this compartment, NK cells release IFNγ (directly promoting the development of Th1 responses) and exert their cytotoxic activity against different types of targets, including tumor cells or (in an allogeneic system such as in transplantation) other target cells including patient’s DCs (46).

In line with these data, Martin-Fontecha et al. showed that murine NK cells can be recruited to lymph nodes in a CXCR3-dependent manner by the injection of mDCs and that these recruitment correlated with the induction of Th1 responses (60). The induction of IFNγ release by NK cells migrated into lymph nodes appears to depend on direct interactions with DCs that release IL-12 and provide IL-15 by trans-presentation (61).

Very recently, it has been shown that NK cells can be genetically reprogramed efficiently using a electroporation method with mRNA coding for the chemokine receptor CCR7, in order to induce the expression of this chemokine receptor on their surface and the consequent NK cell migration toward the lymph node-associated chemokine CCL19 (62).

## Does CCR7^+^ Expression on Alloreactive KIR^+^ NK Cells Plays a Role in Transplantation?

Alloreactivity in haploidentical haplo-HSCT is operating through the mechanism of “missing self” recognition, provided that the donor expresses a given inhibitory KIR, whose ligand is missing in the recipient’s HLA genotype. This combination will lead to a KIR/KIR–ligand mismatch in graft-versus-host (GvH) direction (63–67). In particular, it has been shown that, in T-cell depleted haplo-HSCT, “alloreactive” NK cells kill KIR–ligand mismatched leukemic blasts (graft versus leukemia, GvL), thus contributing to eradication of high-risk acute myeloid or lymphoid leukemias. It is of note that, in this transplantation setting, all cases would be at risk of T cell-mediated alloreactivity both in the HvG and in the GvH direction. However, patients transplanted from an NK “alloreactive donor” benefit from higher rates of engraftment and reduced incidence of graft-versus-host disease (GvHD) (68–71).

Such low rate of GvHD would be consequent to the inefficient priming of alloreactive donor T cells (reconstituted from donor CD34^+^ HSC precursors) due to the NK cell-mediated killing of recipient’s APCs (68). Furthermore, NK cells are able to eliminate residual patient’s T cells, thus preventing HvG reactions and thereby promoting engraftment. In agreement with this concept, *in vitro* studies showed that alloreactive NK cell clones are able to kill not only leukemic blasts (71) but also allogeneic mDCs and T cell blasts (59, 72), while sparing other tissues that are common targets for T-cell-mediated GvHD (70). Although some of these target cells might be killed within peripheral tissues, it is conceivable that the elimination of patient’s DCs by alloreactive NK cells occurs primarily within lymph nodes, thus preventing GvH reactions. Indeed, it is mainly at these sites that patient’s DCs would prime donor’s allospecific naïve T cells.

However, since KIR^+^ NK cells do not express CCR7, it was difficult to explain how donor’s KIR-mismatched NK cells could get in close proximity with recipient DCs and kill them within lymph nodes. In this context, our findings on trogocytosis of CCR7 have provided a possible explanation for the mechanism by which KIR-mismatched NK cells can acquire CCR7, migrate to lymph nodes, and kill recipient’s DC, thus preventing GvH responses in this compartment (52).

Recent evidence revealed that the expression of activating KIRs, in particular KIR2DS1 (specific for HLA-C2) could improve the clinical outcome in haplo-HSCT. It is interesting that the expression of KIR2DS1 can induce alloreactivity by an otherwise non-alloreactive subset of NK cells expressing NKG2A. Thus, in appropriate donor/recipient pairs, the expression of KIR2DS1 can considerably increase the fraction of alloreactive NK cells (71, 72). In light of these data, we could demonstrate that the activating KIR KIR2DS1 represented an advantage for CCR7^−^ NK cells favoring the acquisition of this receptor from HLA-C2^+^ CCR7^+^ cells. In particular, we showed that NK cell, triggered by this receptor in NK cell clones expressing the KIR2DS1^+^/NKG2A^+^ phenotype (isolated from C1^+^ donors), could by-pass almost completely the inhibition mediated by NKG2A, thus making NKG2A^+^ cells (which usually are not alloreactive) capable of killing allogeneic target cells expressing HLA-C2^+^ and HLA-E. Remarkably, this interaction results also in the capture of CCR7 from CCR7^+^ cells that express HLA-C2, including EBV-transformed B cell lines as well as mDCs or T blasts derived from C2^+^ donors. We also showed that the ability to migrate in response to CCL19/21 NK cell was directly proportional to the level of expression of CCR7 acquired after coculture with CCR7^+^ cells (54). Hence, the acquisition of CCR7 on CD56^dim^ KIR^+^ NK cells is negatively regulated by inhibitory KIRs, but is strongly favored by positive signals delivered by activating KIR (52, 54, 59). In line with these results, it has been shown that also the KIR2DS4-activating receptor (73, 74) may induce the uptake of CCR7 by some KIR2DS4^+^ NKG2A^+^ NK cell clones upon interaction with CCR7^+^ target cells expressing appropriate HLA-C alleles (51).

In conclusion, it is conceivable that the migration of CD56^dim^ KIR^+^ NK cells to lymph nodes may play an important role not only in the mechanism of Th1 polarization in adaptive immune responses (through the release of IFNγ and the mechanism of DC “editing”) but also in the prevention of GvHD and HvGD in haplo-HSCT.

## Are There Potential Clinical Application Suggested by These Findings?

**Figure 2:**
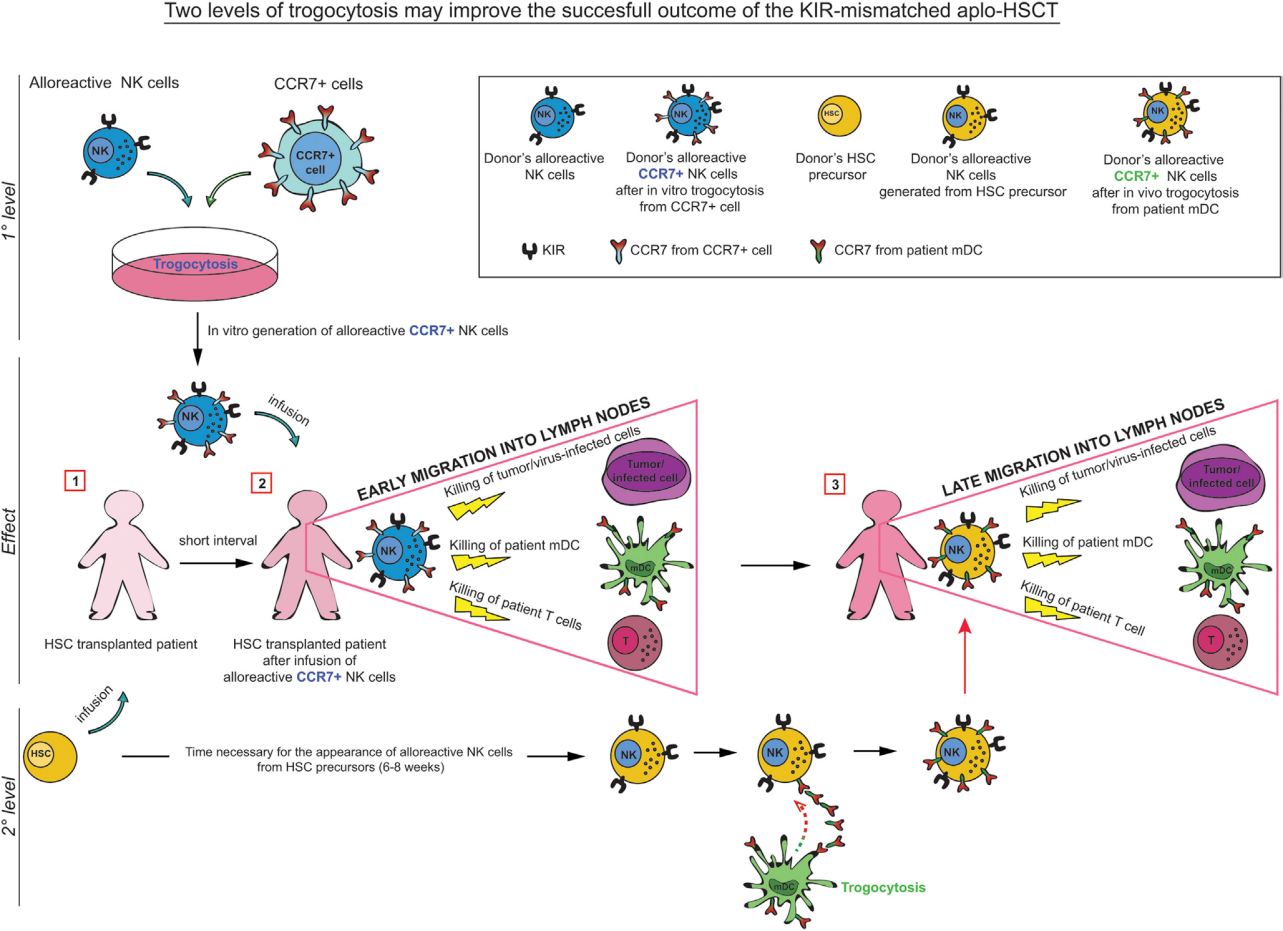
**In KIR-mismatched haplo-HSCT, the late appearance of alloreactive (KIR^+^) NK cells (6–8 weeks after transplant) may result in a delayed anti-leukemia and antiviral infection effect**. In order to prevent these risks, a promising approach has been proposed consisting in the infusion of manipulated NK cells at short time intervals after HSCT. In particular, trogocytosis could be used as a tool to rapidly and transiently modify the migratory properties of donor-derived alloreactive NK cells by inducing CCR7 expression on their surface. These alloreactive NK cells, infused during the time required for the maturation of NK cells from HSC, may play a relevant role in preventing leukemic relapse (GvL activity), GvH/HvG reactions (by killing patient’s DCs/Tcells), and in controlling infections. After this period, mature alloreactive NK cells generated from HSC acquire CCR7 directly (*in vivo*) from patient’s CCR7^+^ cells, including mDC and T cell blasts.

The data described further support the concept that alloreactive NK cells, appropriately selected according to their phenotypic and functional characteristics, may play a central role in haplo-HSCT, by preventing leukemia relapses and improving engraftment by killing leukemia blasts and host APCs, which are known to initiate T cell-mediated GvHD (Figure 2). Notably, in the haplo-HSCT setting, the differentiation of KIR^+^ alloreactive NK cells from CD34^+^ HSC precursors may require 6–8 weeks. Therefore, their anti-leukemia effect would occur only after this time period. In case of infections or highly proliferating residual leukemia blasts, this delay may represent a major problem, resulting in leukemia relapses. In addition, in haplo-HSCT patients, due to the extensive T-cell depletion, required to prevent GvHD, there is an increased risk of life-threatening infections. With the aim to improve posttransplantation immune reconstitution, several immunotherapeutic approaches have been applied. For example, mature donor alloreactive NK cells may be infused at transplantation together with HSC and/or at short time intervals after HSCT. These NK cells may rapidly kill leukemic cells, thus anticipating the effect of alloreactive NK cells generated from transplanted HSC (75–77).

Another approach that could improve the efficiency of alloreactive NK cells would be to exploit trogocytosis as a tool to rapidly (and transiently) modify lymphocytes, for adoptive immunotherapy applications (52, 54). For example, pre-incubation of KIR^+^ NK cells (from an alloreactive donor) with CCR7^+^ HLA class I-deficient cells, before their adoptive transfer to the patient, would allow CCR7^+^ alloreactive NK cells to be rapidly recruited to lymph nodes and to eliminate the leukemia cells residual after conditioning and patient’s DC/T cells (78) (Figure 2). Importantly, in these immunosuppressed patients, CCR7^+^ KIR^+^ NK cells could also provide a rapid and more efficient line of defense against life-threatening infections (Figure 2).

In this context, CCR7 acquired by NK cells *via* trogocytosis has been shown to enhance their lymph node homing upon adoptive transfer to athymic nude mice, contributing to eliminate lymph node tumor metastases (53).

Trogocytosis may involve several cell surface molecules and different cell types, including T and B lymphocytes, NK cells, DCs, and tumor cells. It has been proposed that the formation of the immune synapse for capturing target cell membrane fragments by NK cells may be promoted by the interaction of NK receptors with their specific ligands. During this process several molecules (included in the immune synapse?) can be transferred from one cell to another. For example, upon coculture with the CCR7^+^ 221 cell line, NK cells may acquire, together with CCR7, also CD19 and CD86 (a marker commonly used to assess trogocytosis) (54).

Another interesting possible approach is based on the use of anti-KIR mAbs (their use is now in phase II clinical trials in patients with acute myeloid leukemia or multiple myeloma) (79–83). By blocking inhibitory KIRs, these mAbs, are able to confer alloreactivity to any KIR-2D^+^ NK cell (in individuals expressing HLA-specific activating KIRs, such as KIR2DS1, it is possible that the use of anti-KIR mAbs may partially modulate the function of an NK cell subset expressing this receptor) (52, 54). Notably, in this setting, all anti-KIR-treated NK cells of a given patient become capable of capturing CCR7 by any autologous CCR7^+^ cell soon after infusion in the patient. These “pseudo-alloreactive” NK cells could be rapidly routed toward lymph nodes, where they could carry out their functions.

## Conclusion

The cross talk occurring between NK cells and DCs further supports the concept that NK cells play a critical role in the initiation and regulation of both innate and adaptive immune responses. These cellular interactions allow the establishment of important processes crucial for shaping of adaptive immunity: (a) the “DC editing program” is a process resulting in the selection of DCs with optimal antigen-presenting properties allowing appropriate Th1 responses protective against tumors and infections; (b) the acquisition of CCR7 expression by human KIR^+^ NK cells allows mature cytolytic NK cells to migrate to lymph nodes and exert antitumor and antiviral activity. Moreover, at these sites, NK cells may modulate Th1 polarization. In addition, in the haplo-HSCT transplantation setting “alloreactive” NK cells, migrated to lymph nodes, can mediate important anti-GvH and anti-HvG responses by killing recipient’s allogeneic cells, including lymph node DCs and T cell blasts.

As illustrated in this review, CCR7 expression by KIR^+^ cytolytic NK cells is based on the capture of this chemokine receptor from allogeneic CCR7^+^ cells by a mechanism termed trogocytosis. This event is induced by activating KIRs, including KIR2DS1 and KIR2DS4, while it is negatively regulated by inhibitory KIRs and NKG2A. Thus, selection of appropriate donor/recipient pairs may greatly expand the contingent of alloreactive NK cells migrating to lymph nodes in HSCT, by increasing the effectiveness of alloreactive NK cells and the positive outcome of transplantation.

In addition to various protocols of chemotherapy and radiotherapy, fundamental progress in fighting cancer have been recently obtained with immunotherapy, thanks to the use of anti-immune checkpoints monoclonal antibodies, such as anti-CTLA4 and anti-PD-1, that are capable of reversing the function of otherwise exhausted tumor-specific T lymphocytes (84–86). Importantly, similar novel strategies now can be used also to manipulate NK cell function by the use of antibodies targeting their major inhibitory checkpoints, such as anti-KIR (79, 82), anti-NKG2A (87), and anti-PD-1 (86). In addition, novel means to optimize NK cell traffic to crucial sites (e.g., CCR7 acquisition and homing to lymph nodes) could be applied to treatment of different diseases besides tumors. These include viral infections and autoimmune diseases. In conclusion, a promising approach to improve the cure of life-threatening diseases may be based on “reshaping” NK cell phenotypic and functional properties.

## Author Contributions

All authors listed have made substantial, direct, and intellectual contribution to the work and approved it for publication.

## Conflict of Interest Statement

AM is founder and shareholder of Innate-Pharma (Marseille, France). The remaining authors have no conflicting financial interests.

## References

[1] Vivier E Raulet DH Moretta A Caligiuri MA Zitvogel L Lanier LL Innate or adaptive immunity? The example of natural killer cells. Science (2011) 331(6013):44–9. 10.1126/Science.1198687 21212348 PMC3089969

[2] Lanier LL NK cell receptors. Annu Rev Immunol (1998) 16:359–93. 10.1146/annurev.immunol.16.1.359 9597134

[3] Sivori S Carlomagno S Pesce S Moretta A Vitale M Marcenaro E. TLR/NCR/KIR: which one to use and when? Front Immunol (2014) 5:105. 10.3389/fimmu.2014.00105 24678311 PMC3958761

[4] Cooper MA Fehniger TA Caligiuri MA. The biology of human natural killer-cell subsets. Trends Immunol (2001) 22(11):633–40. 10.1016/S1471-4906(01)02060-9 11698225

[5] Fauriat C Ivarsson MA Ljunggren HG Malmberg KJ Michaelsson J. Education of human natural killer cells by activating killer cell immunoglobulin-like receptors. Blood (2010) 115(6):1166–74. 10.1182/blood-2009-09-245746 19903900

[6] De Maria A Bozzano F Cantoni C Moretta L. Revisiting human natural killer cell subset function revealed cytolytic CD56(dim)CD16+ NK cells as rapid producers of abundant IFN-gamma on activation. Proc Natl Acad Sci U S A (2011) 108(2):728–32. 10.1073/pnas.1012356108 21187373 PMC3021076

[7] Chan A Hong DL Atzberger A Kollnberger S Filer AD Buckley CD CD56bright human NK cells differentiate into CD56dim cells: role of contact with peripheral fibroblasts. J Immunol (2007) 179(1):89–94. 10.4049/jimmunol.179.1.89 17579025

[8] Bjorkstrom NK Riese P Heuts F Andersson S Fauriat C Ivarsson MA Expression patterns of NKG2A, KIR, and CD57 define a process of CD56dim NK-cell differentiation uncoupled from NK-cell education. Blood (2010) 116(19):3853–64. 10.1182/blood-2010-04-281675 20696944

[9] Della Chiesa M Marcenaro E Sivori S Carlomagno S Pesce S Moretta A. Human NK cell response to pathogens. Semin Immunol (2014) 26(2):152–60. 10.1016/j.smim.2014.02.001 24582551

[10] Moretta L Dissecting CD56dim human NK cells. Blood (2010) 116(19):3689–91. 10.1182/blood-2010-09-303057 21071612

[11] Lysakova-Devine T O’Farrelly C. Tissue-specific NK cell populations and their origin. J Leukoc Biol (2014) 96(6):981–90. 10.1189/jlb.1RU0514-241R 25246601

[12] Shibuya A. Development and functions of natural killer cells. Int J Hematol (2003) 78(1):1–6. 10.1007/BF02983233 12894844

[13] Gregoire C Chasson L Luci C Tomasello E Geissmann F Vivier E The trafficking of natural killer cells. Immunol Rev (2007) 220:169–82. 10.1111/j.1600-065X.2007.00563.x 17979846 PMC7165697

[14] Carrega P Bonaccorsi I Di Carlo E Morandi B Paul P Rizzello V CD56(bright)perforin(low) noncytotoxic human NK cells are abundant in both healthy and neoplastic solid tissues and recirculate to secondary lymphoid organs via afferent lymph. J Immunol (2014) 192(8):3805–15. 10.4049/jimmunol.1301889 24646734

[15] Wald O Weiss ID Wald H Shoham H Bar-Shavit Y Beider K IFN-gamma acts on T cells to induce NK cell mobilization and accumulation in target organs. J Immunol (2006) 176(8):4716–29. 10.4049/jimmunol.176.8.4716 16585565

[16] Shi FD Ljunggren HG La Cava A Van Kaer L. Organ-specific features of natural killer cells. Nat Rev Immunol (2011) 11(10):658–71. 10.1038/nri3065 21941294 PMC3620656

[17] Campbell JJ Qin S Unutmaz D Soler D Murphy KE Hodge MR Unique subpopulations of CD56+ NK and NK-T peripheral blood lymphocytes identified by chemokine receptor expression repertoire. J Immunol (2001) 166(11):6477–82. 10.4049/jimmunol.166.11.6477 11359797

[18] Vitale M Della Chiesa M Carlomagno S Romagnani C Thiel A Moretta L The small subset of CD56brightCD16-natural killer cells is selectively responsible for both cell proliferation and interferon-gamma production upon interaction with dendritic cells. Eur J Immunol (2004) 34(6):1715–22. 10.1002/eji.200425100 15162442

[19] Ferlazzo G Thomas D Lin SL Goodman K Morandi B Muller WA The abundant NK cells in human secondary lymphoid tissues require activation to express killer cell Ig-like receptors and become cytolytic. J Immunol (2004) 172(3):1455–62. 10.4049/jimmunol.172.3.1455 14734722

[20] Parolini S Santoro A Marcenaro E Luini W Massardi L Facchetti F The role of chemerin in the colocalization of NK and dendritic cell subsets into inflamed tissues. Blood (2007) 109(9):3625–32. 10.1182/blood-2006-08-038844 17202316

[21] Frey M Packianathan NB Fehniger TA Ross ME Wang WC Stewart CC Differential expression and function of L-selectin on CD56bright and CD56dim natural killer cell subsets. J Immunol (1998) 161(1):400–8. 9647249

[22] Fehniger TA Cooper MA Nuovo GJ Cella M Facchetti F Colonna M CD56bright natural killer cells are present in human lymph nodes and are activated by T cell-derived IL-2: a potential new link between adaptive and innate immunity. Blood (2003) 101(8):3052–7. 10.1182/blood-2002-09-2876 12480696

[23] Sharma R Das A. Organ-specific phenotypic and functional features of NK cells in humans. Immunol Res (2014) 58(1):125–31. 10.1007/s12026-013-8477-9 24366663

[24] Belisle JA Gubbels JA Raphael CA Migneault M Rancourt C Connor JP Peritoneal natural killer cells from epithelial ovarian cancer patients show an altered phenotype and bind to the tumour marker MUC16 (CA125). Immunology (2007) 122(3):418–29. 10.1111/j.1365-2567.2007.02660.x 17617155 PMC2266014

[25] Carlsten M Norell H Bryceson YT Poschke I Schedvins K Ljunggren HG Primary human tumor cells expressing CD155 impair tumor targeting by down-regulating DNAM-1 on NK cells. J Immunol (2009) 183(8):4921–30. 10.4049/jimmunol.0901226 19801517

[26] Pesce S Tabellini G Cantoni C Patrizi O Coltrini D Rampinelli F B7-H6-mediated downregulation of NKp30 in NK cells contributes to ovarian carcinoma immune escape. Oncoimmunology (2015) 4(4):e1001224. 10.1080/2162402X.2014.1001224 26137398 PMC4485754

[27] Ali TH Pisanti S Ciaglia E Mortarini R Anichini A Garofalo C Enrichment of CD56(dim)KIR + CD57 + highly cytotoxic NK cells in tumour-infiltrated lymph nodes of melanoma patients. Nat Commun (2014) 5:5639. 10.1038/ncomms6639 25472612 PMC4338526

[28] Moretta A. Natural killer cells and dendritic cells: rendezvous in abused tissues. Nat Rev Immunol (2002) 2(12):957–64. 10.1038/nri956 12461568

[29] Zitvogel L Dendritic and natural killer cells cooperate in the control/switch of innate immunity. J Exp Med (2002) 195(3):F9–14. 10.1084/jem.20012040 11828015 PMC2193597

[30] Cooper MA Fehniger TA Fuchs A Colonna M Caligiuri MA. NK cell and DC interactions. Trends Immunol (2004) 25(1):47–52. 10.1016/j.it.2003.10.012 14698284

[31] Marcenaro E Ferranti B Moretta A. NK-DC interaction: on the usefulness of auto-aggression. Autoimmun Rev (2005) 4(8):520–5. 10.1016/j.autrev.2005.04.015 16214089

[32] Moretta A Bottino C Vitale M Pende D Cantoni C Mingari MC Activating receptors and coreceptors involved in human natural killer cell-mediated cytolysis. Annu Rev Immunol (2001) 19:197–223. 10.1146/annurev.immunol.19.1.197 11244035

[33] Sivori S Falco M Della Chiesa M Carlomagno S Vitale M Moretta L CpG and double-stranded RNA trigger human NK cells by toll-like receptors: induction of cytokine release and cytotoxicity against tumors and dendritic cells. Proc Natl Acad Sci U S A (2004) 101(27):10116–21. 10.1073/pnas.0403744101 15218108 PMC454174

[34] Marcenaro E Ferranti B Falco M Moretta L Moretta A. Human NK cells directly recognize *Mycobacterium bovis* via TLR2 and acquire the ability to kill monocyte-derived DC. Int Immunol (2008) 20(9):1155–67. 10.1093/intimm/dxn073 18596023

[35] Marcenaro E Della Chiesa M Bellora F Parolini S Millo R Moretta L IL-12 or IL-4 prime human NK cells to mediate functionally divergent interactions with dendritic cells or tumors. J Immunol (2005) 174(7):3992–8. 10.4049/jimmunol.174.7.3992 15778356

[36] Della Chiesa M Vitale M Carlomagno S Ferlazzo G Moretta L Moretta A. The natural killer cell-mediated killing of autologous dendritic cells is confined to a cell subset expressing CD94/NKG2A, but lacking inhibitory killer Ig-like receptors. Eur J Immunol (2003) 33(6):1657–66. 10.1002/eji.200390042 12778484

[37] Ferlazzo G Moretta L. Dendritic cell editing by natural killer cells. Crit Rev Oncog (2014) 19(1–2):67–75. 10.1615/CritRevOncog.2014010827 24941374

[38] Ferlazzo G Morandi B D’Agostino A Meazza R Melioli G Moretta A The interaction between NK cells and dendritic cells in bacterial infections results in rapid induction of NK cell activation and in the lysis of uninfected dendritic cells. Eur J Immunol (2003) 33(2):306–13. 10.1002/immu.200310004 12548561

[39] Sahoo A Wali S Nurieva R T helper 2 and T follicular helper cells: regulation and function of interleukin-4. Cytokine Growth Factor Rev (2016) 30:29–37. 10.1016/j.cytogfr.2016.03.011 27072069 PMC5110032

[40] Waggoner SN Reighard SD Gyurova IE Cranert SA Mahl SE Karmele EP Roles of natural killer cells in antiviral immunity. Curr Opin Virol (2016) 16:15–23. 10.1016/j.coviro.2015.10.008 26590692 PMC4821726

[41] Agaugue S Marcenaro E Ferranti B Moretta L Moretta A Human natural killer cells exposed to IL-2, IL-12, IL-18, or IL-4 differently modulate priming of naive T cells by monocyte-derived dendritic cells. Blood (2008) 112(5):1776–83. 10.1182/blood-2008-02-135871 18579793

[42] Ferlazzo G Munz C NK cell compartments and their activation by dendritic cells. J Immunol (2004) 172(3):1333–9. 10.4049/jimmunol.172.3.1333 14734707

[43] Gerosa F Baldani-Guerra B Nisii C Marchesini V Carra G Trinchieri G. Reciprocal activating interaction between natural killer cells and dendritic cells. J Exp Med (2002) 195(3):327–33. 10.1084/jem.20010938 11828007 PMC2193595

[44] Piccioli D Sbrana S Melandri E Valiante NM. Contact-dependent stimulation and inhibition of dendritic cells by natural killer cells. J Exp Med (2002) 195(3):335–41. 10.1084/jem.20010934 11828008 PMC2193592

[45] Moretta A Marcenaro E Parolini S Ferlazzo G Moretta L NK cells at the interface between innate and adaptive immunity. Cell Death Differ (2008) 15(2):226–33. 10.1038/sj.cdd.4402170 17541426

[46] Moretta L Locatelli F Pende D Marcenaro E Mingari MC Moretta A. Killer Ig-like receptor-mediated control of natural killer cell alloreactivity in haploidentical hematopoietic stem cell transplantation. Blood (2011) 117(3):764–71. 10.1182/blood-2010-08-264085 20889923

[47] Mailliard RB Alber SM Shen H Watkins SC Kirkwood JM Herberman RB IL-18-induced CD83+CCR7+ NK helper cells. J Exp Med (2005) 202(7):941–53. 10.1084/jem.20050128 16203865 PMC2213172

[48] Bellora F Castriconi R Dondero A Reggiardo G Moretta L Mantovani A The interaction of human natural killer cells with either unpolarized or polarized macrophages results in different functional outcomes. Proc Natl Acad Sci U S A (2010) 107(50):21659–64. 10.1073/pnas.1007654108 21118979 PMC3003022

[49] Bajenoff M Glaichenhaus N Germain RN. Fibroblastic reticular cells guide T lymphocyte entry into and migration within the splenic T cell zone. J Immunol (2008) 181(6):3947–54. 10.4049/jimmunol.181.6.3947 18768849 PMC2596721

[50] White MJ Nielsen CM McGregor RH Riley EH Goodier MR. Differential activation of CD57-defined natural killer cell subsets during recall responses to vaccine antigens. Immunology (2014) 142(1):140–50. 10.1111/imm.12239 24843874 PMC3992055

[51] Pesce S Carlomagno S Moretta A Sivori S Marcenaro E Uptake of CCR7 by KIR2DS4(+) NK cells is induced upon recognition of certain HLA-C alleles. J Immunol Res (2015) 2015:754373 10.1155/2015/754373 25961063 PMC4415677

[52] Marcenaro E Cantoni C Pesce S Prato C Pende D Agaugue S Uptake of CCR7 and acquisition of migratory properties by human KIR+ NK cells interacting with monocyte-derived DC or EBV cell lines: regulation by KIR/HLA-class I interaction. Blood (2009) 114(19):4108–16. 10.1182/blood-2009-05-222265 19749090

[53] Somanchi SS Somanchi A Cooper LJ Lee DA. Engineering lymph node homing of ex vivo-expanded human natural killer cells via trogocytosis of the chemokine receptor CCR7. Blood (2012) 119(22):5164–72. 10.1182/blood-2011-11-389924 22498742 PMC3418772

[54] Marcenaro E Pesce S Sivori S Carlomagno S Moretta L Moretta A. KIR2DS1-dependent acquisition of CCR7 and migratory properties by human NK cells interacting with allogeneic HLA-C2+ DCs or T-cell blasts. Blood (2013) 121(17):3396–401. 10.1182/blood-2012-09-458752 23449637

[55] Joly E Hudrisier D What is trogocytosis and what is its purpose? Nat Immunol (2003) 4(9):815 10.1038/ni0903-815 12942076

[56] Davis DM. Intercellular transfer of cell-surface proteins is common and can affect many stages of an immune response. Nat Rev Immunol (2007) 7(3):238–43. 10.1038/nri2020 17290299

[57] Mack M Kleinschmidt A Bruhl H Klier C Nelson PJ Cihak J Transfer of the chemokine receptor CCR5 between cells by membrane-derived microparticles: a mechanism for cellular human immunodeficiency virus 1 infection. Nat Med (2000) 6(7):769–75. 10.1038/77498 10888925

[58] Hudrisier D Riond J Mazarguil H Gairin JE Joly E. Cutting edge: CTLs rapidly capture membrane fragments from target cells in a TCR signaling-dependent manner. J Immunol (2001) 166(6):3645–9. 10.4049/jimmunol.166.6.3645 11238601

[59] Marcenaro E Carlomagno S Pesce S Della Chiesa M Moretta A Sivori S. Role of alloreactive KIR2DS1(+) NK cells in haploidentical hematopoietic stem cell transplantation. J Leukoc Biol (2011) 90(4):661–7. 10.1189/jlb.0311137 21791599

[60] Martin-Fontecha A Thomsen LL Brett S Gerard C Lipp M Lanzavecchia A Induced recruitment of NK cells to lymph nodes provides IFN-gamma for T(H)1 priming. Nat Immunol (2004) 5(12):1260–5. 10.1038/ni1138 15531883

[61] Lucas M Schachterle W Oberle K Aichele P Diefenbach A. Dendritic cells prime natural killer cells by trans-presenting interleukin 15. Immunity (2007) 26(4):503–17. 10.1016/j.immuni.2007.03.006 17398124 PMC2084390

[62] Carlsten M Levy E Karambelkar A Li L Reger R Berg M Efficient mRNA-based genetic engineering of human NK cells with high-affinity CD16 and CCR7 augments rituximab-induced ADCC against lymphoma and targets NK cell migration toward the lymph node-associated chemokine CCL19. Front Immunol (2016) 7:105. 10.3389/fimmu.2016.00105 27047492 PMC4801851

[63] Moretta A Bottino C Vitale M Pende D Biassoni R Mingari MC Receptors for HLA class-I molecules in human natural killer cells. Annu Rev Immunol (1996) 14:619–48. 10.1146/annurev.immunol.14.1.619 8717527

[64] Long EO. Regulation of immune responses through inhibitory receptors. Annu Rev Immunol (1999) 17:875–904. 10.1146/annurev.immunol.17.1.875 10358776

[65] Karre K Natural killer cell recognition of missing self. Nat Immunol (2008) 9(5):477–80. 10.1038/ni0508-477 18425103

[66] Moretta A Bottino C Pende D Tripodi G Tambussi G Viale O Identification of four subsets of human CD3-CD16+ natural killer (NK) cells by the expression of clonally distributed functional surface molecules: correlation between subset assignment of NK clones and ability to mediate specific alloantigen recognition. J Exp Med (1990) 172(6):1589–98. 10.1084/jem.172.6.1589 2147946 PMC2188758

[67] Moretta A Vitale M Bottino C Orengo AM Morelli L Augugliaro R P58 molecules as putative receptors for major histocompatibility complex (MHC) class I molecules in human natural killer (NK) cells. Anti-p58 antibodies reconstitute lysis of MHC class I-protected cells in NK clones displaying different specificities. J Exp Med (1993) 178(2):597–604. 10.1084/jem.178.2.597 8340759 PMC2191136

[68] Velardi A Ruggeri L Moretta A Moretta L NK cells: a lesson from mismatched hematopoietic transplantation. Trends Immunol (2002) 23(9):438–44. 10.1016/S1471-4906(02)02284-6 12200065

[69] Ruggeri L Mancusi A Capanni M Urbani E Carotti A Aloisi T Donor natural killer cell allorecognition of missing self in haploidentical hematopoietic transplantation for acute myeloid leukemia: challenging its predictive value. Blood (2007) 110(1):433–40. 10.1182/blood-2006-07-038687 17371948 PMC1896125

[70] Moretta A Locatelli F Moretta L. Human NK cells: from HLA class I-specific killer Ig-like receptors to the therapy of acute leukemias. Immunol Rev (2008) 224:58–69. 10.1111/j.1600-065X.2008.00651.x 18759920

[71] Pende D Marcenaro S Falco M Martini S Bernardo ME Montagna D Anti-leukemia activity of alloreactive NK cells in KIR ligand-mismatched haploidentical HSCT for pediatric patients: evaluation of the functional role of activating KIR and redefinition of inhibitory KIR specificity. Blood (2009) 113(13):3119–29. 10.1182/blood-2008-06-164103 18945967

[72] Sivori S Carlomagno S Falco M Romeo E Moretta L Moretta A. Natural killer cells expressing the KIR2DS1-activating receptor efficiently kill T-cell blasts and dendritic cells: implications in haploidentical HSCT. Blood (2011) 117(16):4284–92. 10.1182/blood-2010-10-316125 21355085

[73] Bottino C Sivori S Vitale M Cantoni C Falco M Pende D A novel surface molecule homologous to the p58/p50 family of receptors is selectively expressed on a subset of human natural killer cells and induces both triggering of cell functions and proliferation. Eur J Immunol (1996) 26(8):1816–24. 10.1002/eji.1830260823 8765026

[74] Katz G Gazit R Arnon TI Gonen-Gross T Tarcic G Markel G MHC class I-independent recognition of NK-activating receptor KIR2DS4. J Immunol (2004) 173(3):1819–25. 10.4049/jimmunol.173.3.1819 15265913

[75] Handgretinger R. New approaches to graft engineering for haploidentical bone marrow transplantation. Semin Oncol (2012) 39(6):664–73. 10.1053/j.seminoncol.2012.09.007 23206843

[76] Locatelli F Moretta F Brescia L Merli P. Natural killer cells in the treatment of high-risk acute leukaemia. Semin Immunol (2014) 26(2):173–9. 10.1016/j.smim.2014.02.004 24613727

[77] Lucarelli B Merli P Bertaina V Locatelli F. Strategies to accelerate immune recovery after allogeneic hematopoietic stem cell transplantation. Expert Rev Clin Immunol (2016) 12(3):343–58. 10.1586/1744666X.2016.1123091 26588325

[78] Locatelli F Pende D Mingari MC Bertaina A Falco M Moretta A Cellular and molecular basis of haploidentical hematopoietic stem cell transplantation in the successful treatment of high-risk leukemias: role of alloreactive NK cells. Front Immunol (2013) 4:15. 10.3389/fimmu.2013.00015 23378843 PMC3561663

[79] Romagne F Andre P Spee P Zahn S Anfossi N Gauthier L Preclinical characterization of 1-7F9, a novel human anti-KIR receptor therapeutic antibody that augments natural killer-mediated killing of tumor cells. Blood (2009) 114(13):2667–77. 10.1182/blood-2009-02-206532 19553639 PMC2756126

[80] Benson DM Jr Hofmeister CC Padmanabhan S Suvannasankha A Jagannath S Abonour R A phase 1 trial of the anti-KIR antibody IPH2101 in patients with relapsed/refractory multiple myeloma. Blood (2012) 120(22):4324–33. 10.1182/blood-2012-06-438028 23033266 PMC3507143

[81] Kohrt HE Thielens A Marabelle A Sagiv-Barfi I Sola C Chanuc F Anti-KIR antibody enhancement of anti-lymphoma activity of natural killer cells as monotherapy and in combination with anti-CD20 antibodies. Blood (2014) 123(5):678–86. 10.1182/blood-2013-08-519199 24326534 PMC3907754

[82] Vey N Bourhis JH Boissel N Bordessoule D Prebet T Charbonnier A A phase 1 trial of the anti-inhibitory KIR mAb IPH2101 for AML in complete remission. Blood (2012) 120(22):4317–23. 10.1182/blood-2012-06-437558 23002117

[83] Nijhof IS Lammerts van Bueren JJ van Kessel B Andre P Morel Y Lokhorst HM Daratumumab-mediated lysis of primary multiple myeloma cells is enhanced in combination with the human anti-KIR antibody IPH2102 and lenalidomide. Haematologica (2015) 100(2):263–8. 10.3324/haematol.2014.117531 25510242 PMC4803142

[84] Lipson EJ Sharfman WH Drake CG Wollner I Taube JM Anders RA Durable cancer regression off-treatment and effective reinduction therapy with an anti-PD-1 antibody. Clin Cancer Res (2013) 19(2):462–8. 10.1158/1078-0432.CCR-12-2625 23169436 PMC3548952

[85] Okazaki T Chikuma S Iwai Y Fagarasan S Honjo T A rheostat for immune responses: the unique properties of PD-1 and their advantages for clinical application. Nat Immunol (2013) 14(12):1212–8. 10.1038/ni.2762 24240160

[86] Pesce S Greppi M Tabellini G Rampinelli F Parolini S Olive D Identification of a subset of human natural killer cells expressing high levels of programmed death 1: a phenotypic and functional characterization. J Allergy Clin Immunol (2016). 10.1016/j.jaci.2016.04.025 27372564

[87] Ruggeri L Urbani E Andre P Mancusi A Tosti A Topini F Effects of anti-NKG2A antibody administration on leukemia and normal hematopoietic cells. Haematologica (2016) 101(5):626–33. 10.3324/haematol.2015.135301 26721894 PMC5004363

